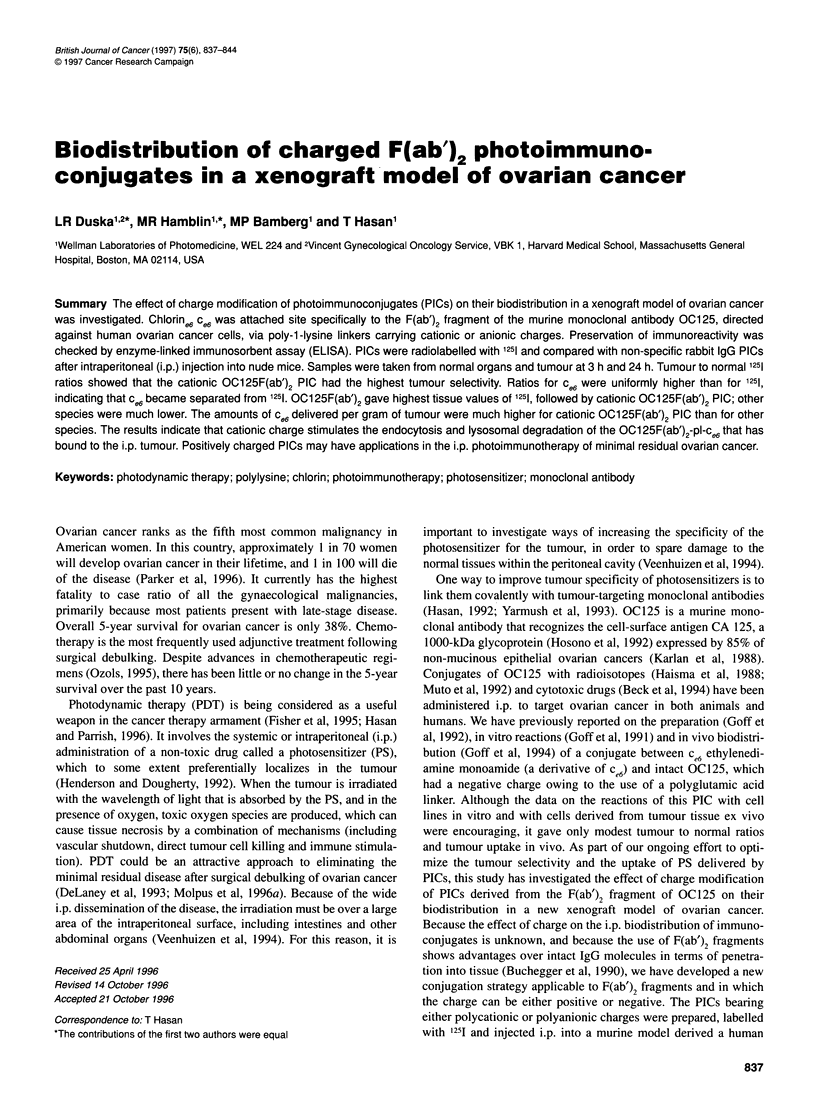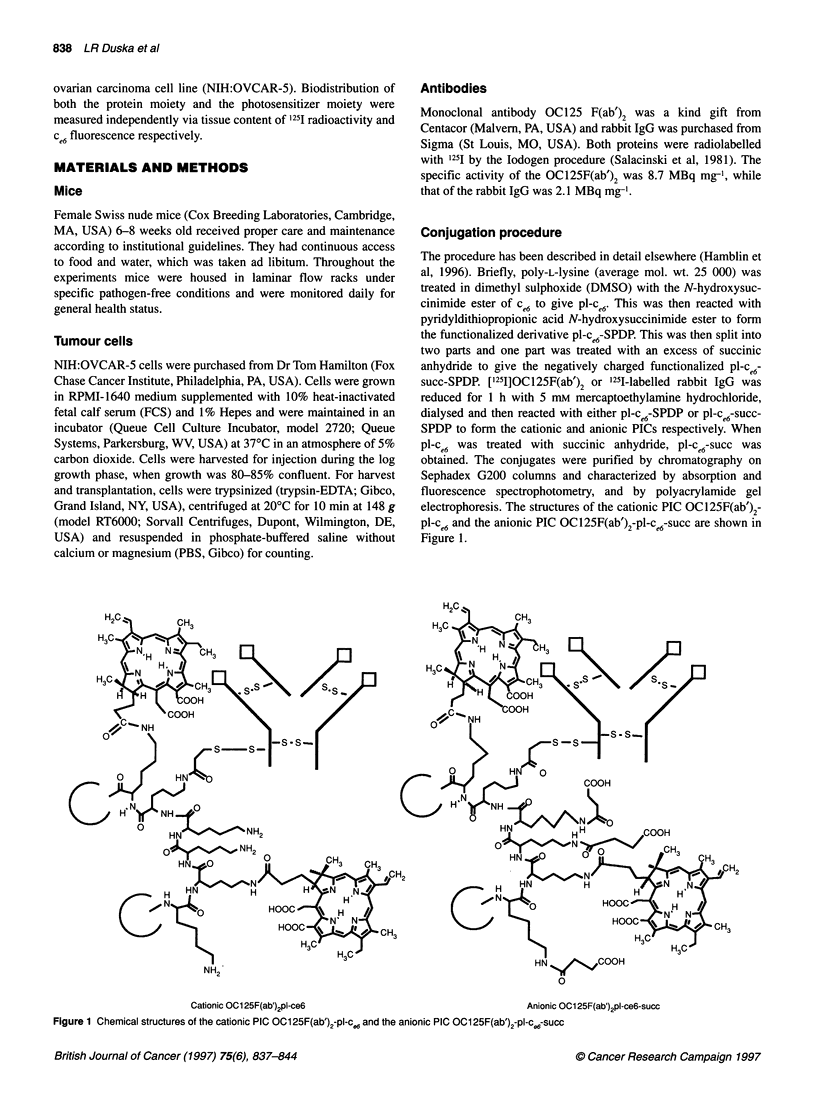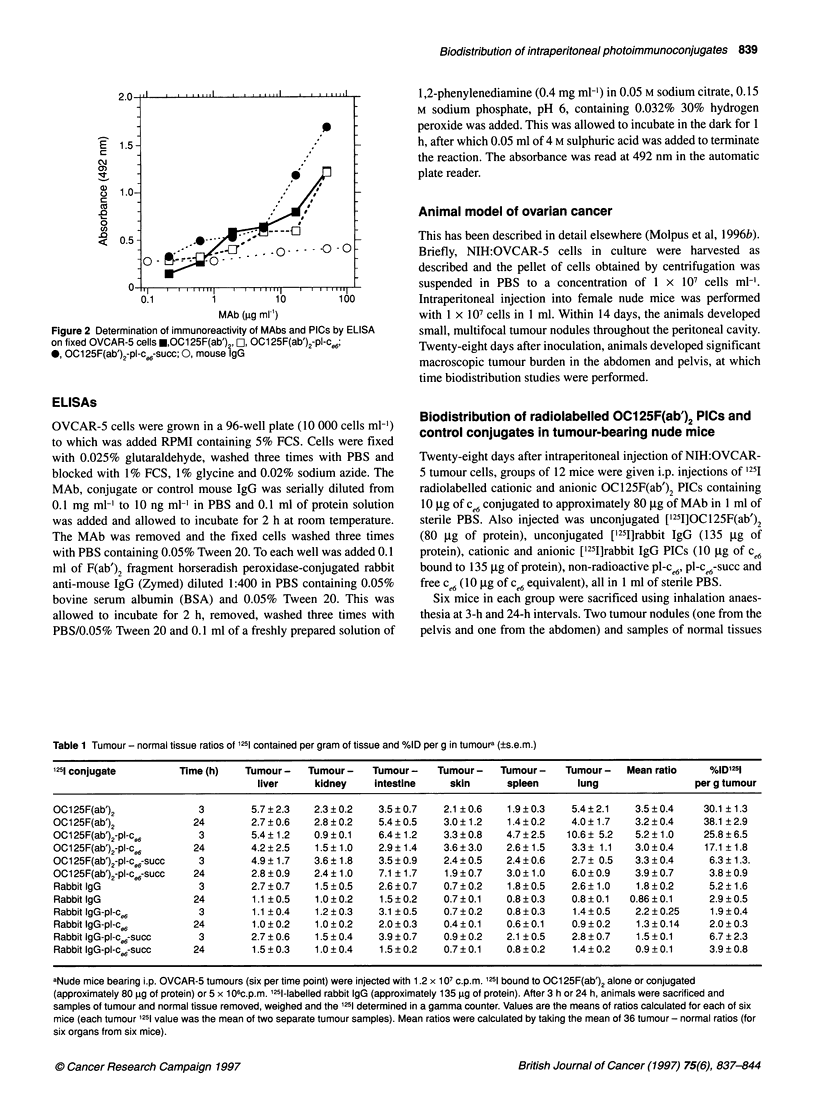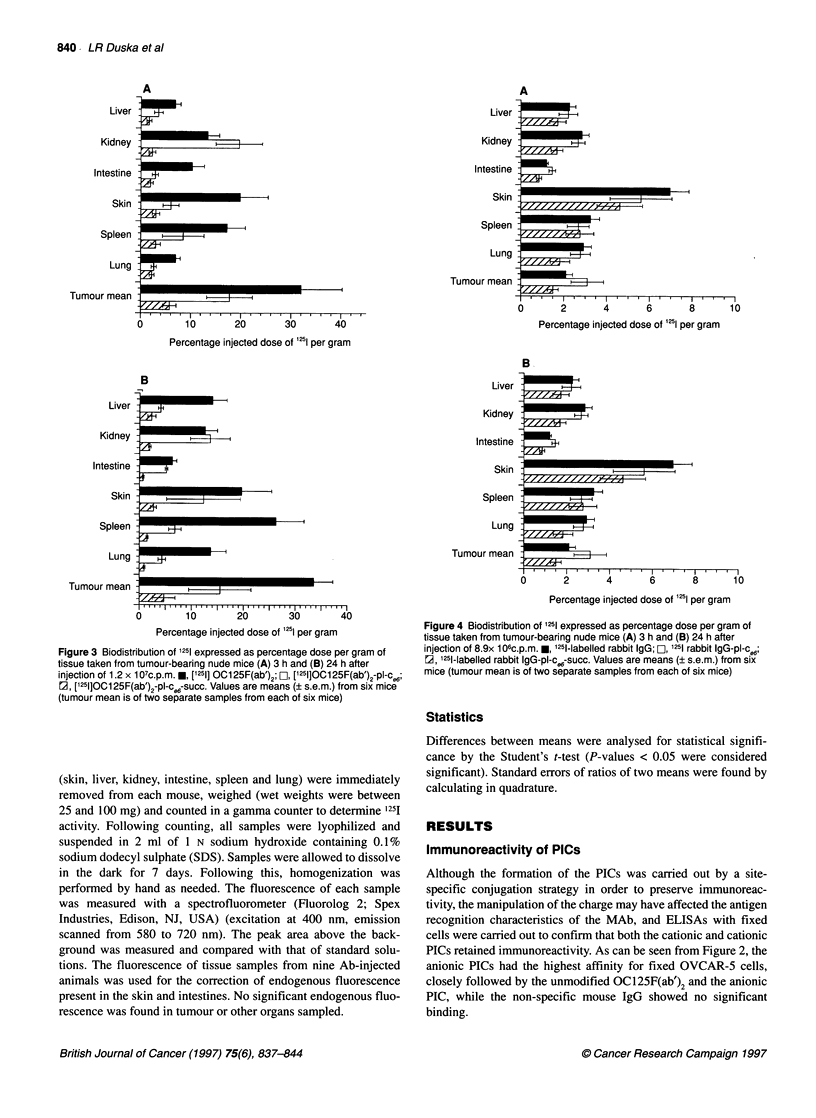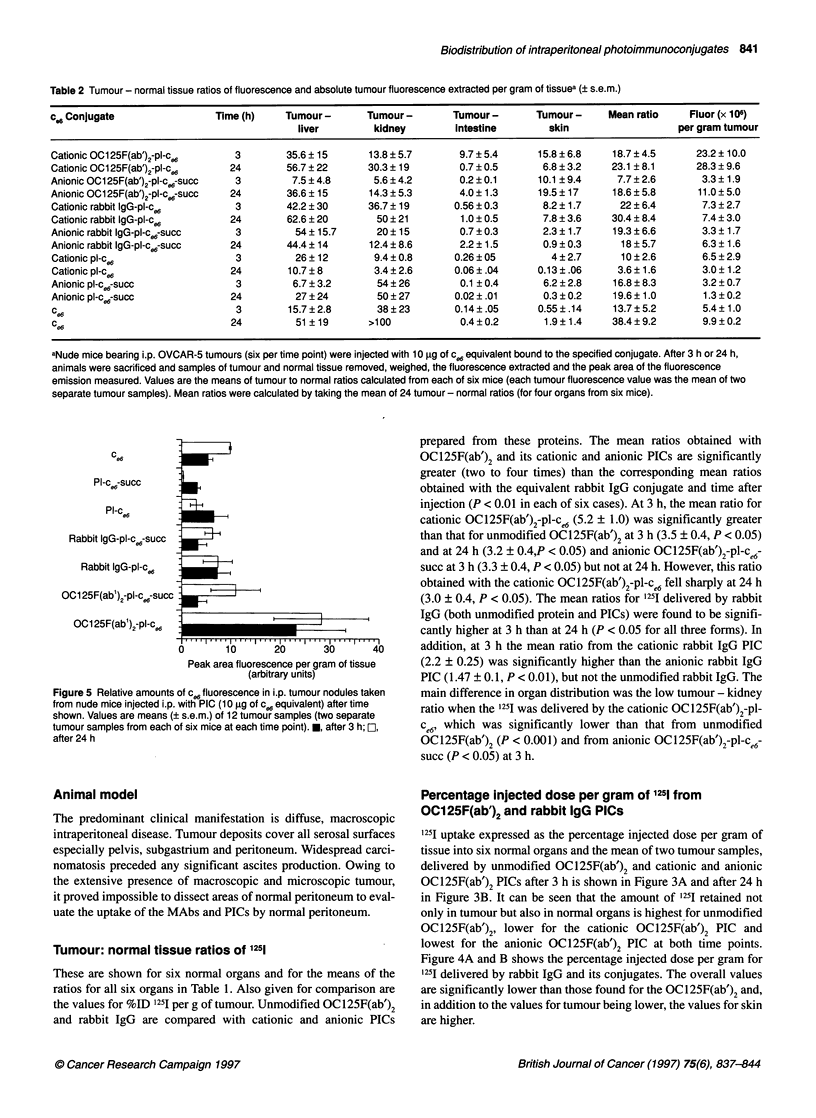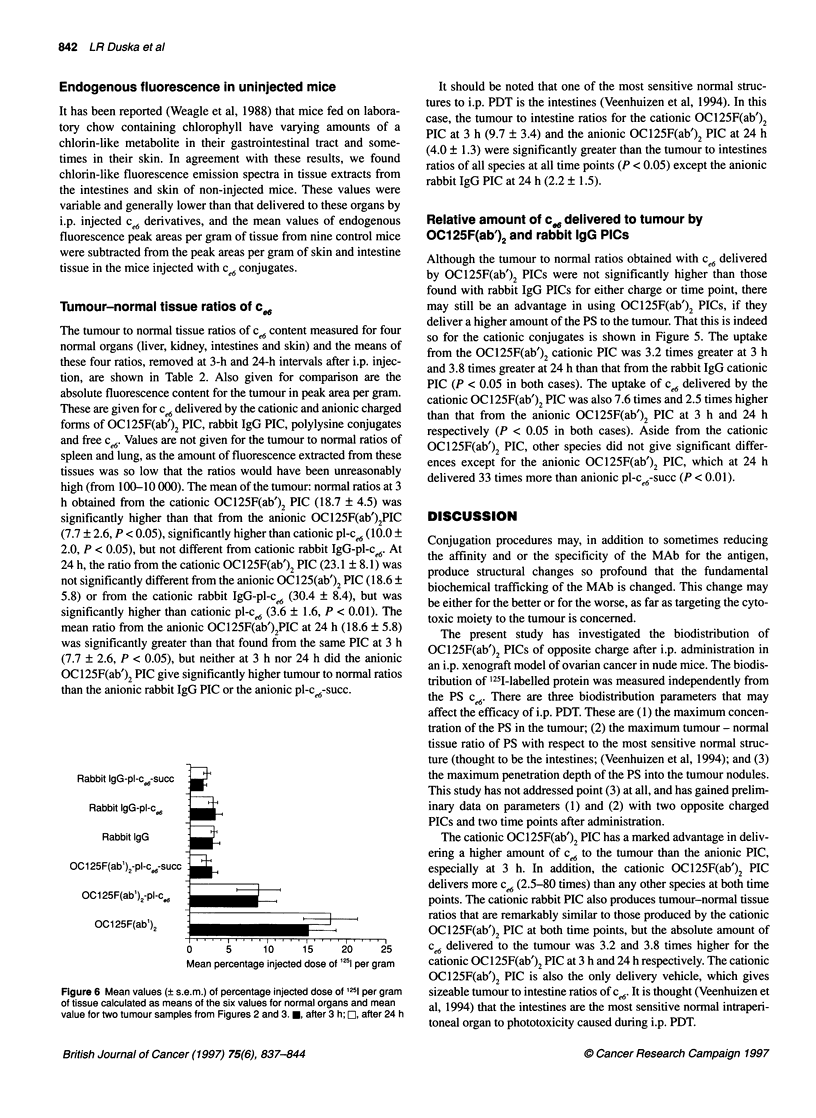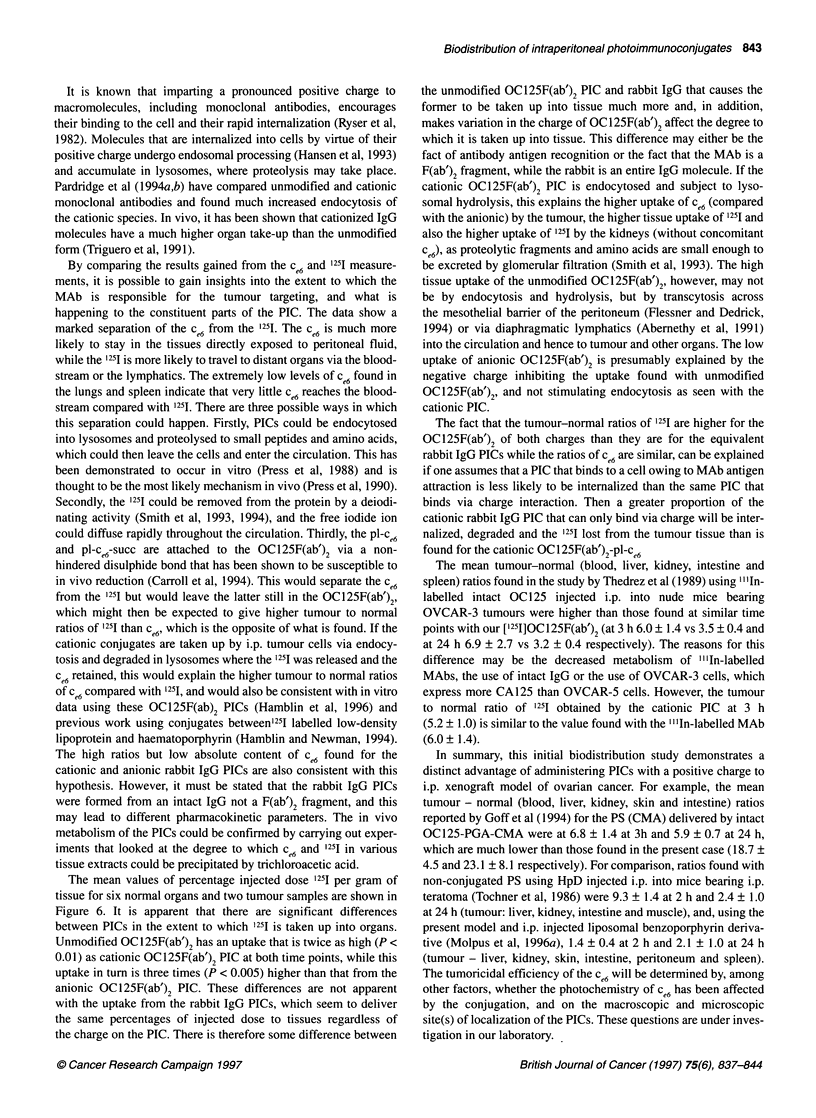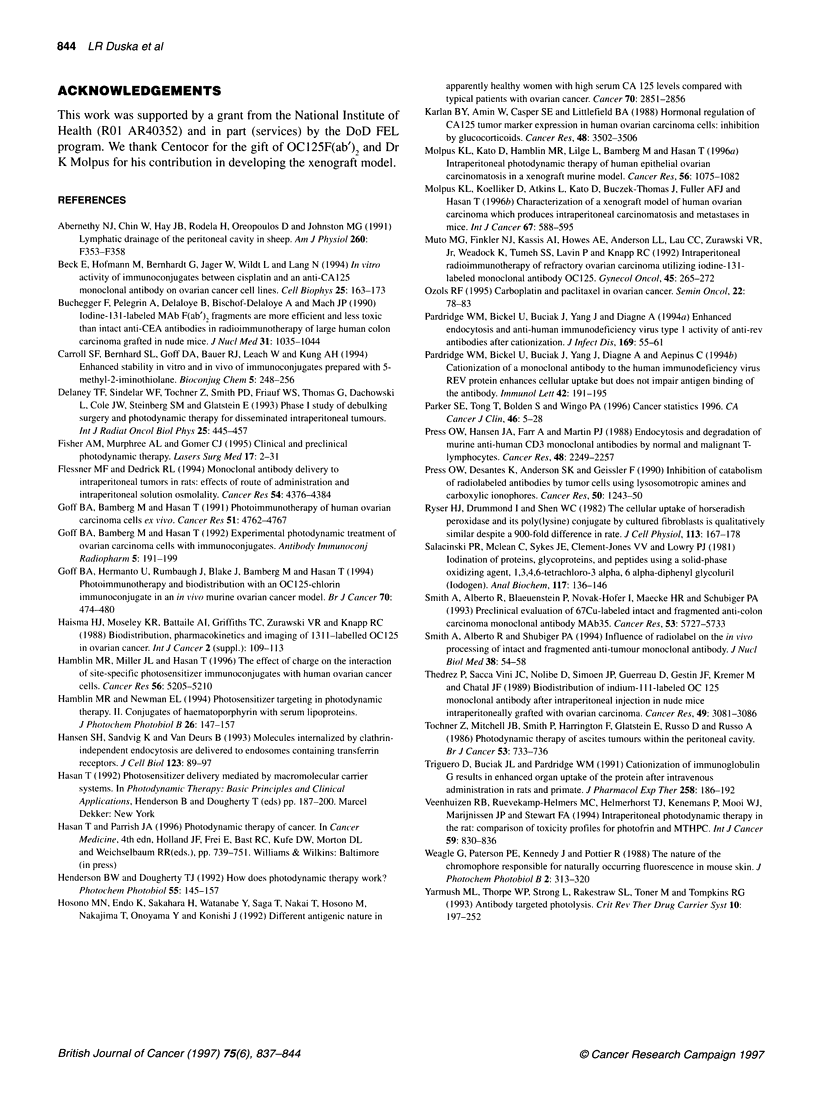# Biodistribution of charged F(ab')2 photoimmunoconjugates in a xenograft model of ovarian cancer.

**DOI:** 10.1038/bjc.1997.149

**Published:** 1997

**Authors:** L. R. Duska, M. R. Hamblin, M. P. Bamberg, T. Hasan

**Affiliations:** Wellman Laboratories of Photomedicine, Harvard Medical School, Masssachusetts General Hospital, Boston 02114, USA.

## Abstract

The effect of charge modification of photoimmunoconjugates (PICs) on their biodistribution in a xenograft model of ovarian cancer was investigated. Chlorin(e6)c(e6) was attached site specifically to the F(ab')2 fragment of the murine monoclonal antibody OC125, directed against human ovarian cancer cells, via poly-1-lysine linkers carrying cationic or anionic charges. Preservation of immunoreactivity was checked by enzyme-linked immunosorbent assay (ELISA). PICs were radiolabelled with 125I and compared with non-specific rabbit IgG PICs after intraperitoneal (i.p.) injection into nude mice. Samples were taken from normal organs and tumour at 3 h and 24 h. Tumour to normal 125I ratios showed that the cationic OC125F(ab')2 PIC had the highest tumour selectivity. Ratios for c(e6) were uniformly higher than for 125I, indicating that c(e6) became separated from 125I. OC125F(ab')2 gave highest tissue values of 125I, followed by cationic OC125F(ab')2 PIC; other species were much lower. The amounts of c(e6) delivered per gram of tumour were much higher for cationic OC125F(ab')2 PIC than for other species. The results indicate that cationic charge stimulates the endocytosis and lysosomal degradation of the OC125F(ab')2-pl-c(e6) that has bound to the i.p. tumour. Positively charged PICs may have applications in the i.p. photoimmunotherapy of minimal residual ovarian cancer.


British Journal of Cancer (1997) 75(6), 837–844
© 1997 Cancer Research Campaign

### Biodistribution of charged F(ab′)$_2$ photoimmunoconjugates in a xenograft model of ovarian cancer

**LR Duska[1,2*], MR Hamblin[1,*], MP Bamberg[1] and T Hasan[1]**

[1]Wellman Laboratories of Photomedicine, WEL 224 and [2]Vincent Gynecological Oncology Service, VBK 1, Harvard Medical School, Massachusetts General Hospital, Boston, MA 02114, USA

**Summary** The effect of charge modification of photoimmunoconjugates (PICs) on their biodistribution in a xenograft model of ovarian cancer was investigated. Chlorin$_{e6}$ $c_{e6}$ was attached site specifically to the F(ab′)$_2$ fragment of the murine monoclonal antibody OC125, directed against human ovarian cancer cells, via poly-1-lysine linkers carrying cationic or anionic charges. Preservation of immunoreactivity was checked by enzyme-linked immunosorbent assay (ELISA). PICs were radiolabelled with $^{125}$I and compared with non-specific rabbit IgG PICs after intraperitoneal (i.p.) injection into nude mice. Samples were taken from normal organs and tumour at 3 h and 24 h. Tumour to normal $^{125}$I ratios showed that the cationic OC125F(ab′)$_2$ PIC had the highest tumour selectivity. Ratios for $c_{e6}$ were uniformly higher than for $^{125}$I, indicating that $c_{e6}$ became separated from $^{125}$I. OC125F(ab′)$_2$ gave highest tissue values of $^{125}$I, followed by cationic OC125F(ab′)$_2$ PIC; other species were much lower. The amounts of $c_{e6}$ delivered per gram of tumour were much higher for cationic OC125F(ab′)$_2$ PIC than for other species. The results indicate that cationic charge stimulates the endocytosis and lysosomal degradation of the OC125F(ab′)$_2$-pl-$c_{e6}$ that has bound to the i.p. tumour. Positively charged PICs may have applications in the i.p. photoimmunotherapy of minimal residual ovarian cancer.

**Keywords:** photodynamic therapy; polylysine; chlorin; photoimmunotherapy; photosensitizer; monoclonal antibody

Ovarian cancer ranks as the fifth most common malignancy in American women. In this country, approximately 1 in 70 women will develop ovarian cancer in their lifetime, and 1 in 100 will die of the disease (Parker et al, 1996). It currently has the highest fatality to case ratio of all the gynaecological malignancies, primarily because most patients present with late-stage disease. Overall 5-year survival for ovarian cancer is only 38%. Chemotherapy is the most frequently used adjunctive treatment following surgical debulking. Despite advances in chemotherapeutic regimens (Ozols, 1995), there has been little or no change in the 5-year survival over the past 10 years.

Photodynamic therapy (PDT) is being considered as a useful weapon in the cancer therapy armament (Fisher et al, 1995; Hasan and Parrish, 1996). It involves the systemic or intraperitoneal (i.p.) administration of a non-toxic drug called a photosensitizer (PS), which to some extent preferentially localizes in the tumour (Henderson and Dougherty, 1992). When the tumour is irradiated with the wavelength of light that is absorbed by the PS, and in the presence of oxygen, toxic oxygen species are produced, which can cause tissue necrosis by a combination of mechanisms (including vascular shutdown, direct tumour cell killing and immune stimulation). PDT could be an attractive approach to eliminating the minimal residual disease after surgical debulking of ovarian cancer (DeLaney et al, 1993; Molpus et al, 1996*a*). Because of the wide i.p. dissemination of the disease, the irradiation must be over a large area of the intraperitoneal surface, including intestines and other abdominal organs (Veenhuizen et al, 1994). For this reason, it is important to investigate ways of increasing the specificity of the photosensitizer for the tumour, in order to spare damage to the normal tissues within the peritoneal cavity (Veenhuizen et al, 1994).

One way to improve tumour specificity of photosensitizers is to link them covalently with tumour-targeting monoclonal antibodies (Hasan, 1992; Yarmush et al, 1993). OC125 is a murine monoclonal antibody that recognizes the cell-surface antigen CA 125, a 1000-kDa glycoprotein (Hosono et al, 1992) expressed by 85% of non-mucinous epithelial ovarian cancers (Karlan et al, 1988). Conjugates of OC125 with radioisotopes (Haisma et al, 1988; Muto et al, 1992) and cytotoxic drugs (Beck et al, 1994) have been administered i.p. to target ovarian cancer in both animals and humans. We have previously reported on the preparation (Goff et al, 1992), in vitro reactions (Goff et al, 1991) and in vivo biodistribution (Goff et al, 1994) of a conjugate between $c_{e6}$ ethylenediamine monoamide (a derivative of $c_{e6}$) and intact OC125, which had a negative charge owing to the use of a polyglutamic acid linker. Although the data on the reactions of this PIC with cell lines in vitro and with cells derived from tumour tissue ex vivo were encouraging, it gave only modest tumour to normal ratios and tumour uptake in vivo. As part of our ongoing effort to optimize the tumour selectivity and the uptake of PS delivered by PICs, this study has investigated the effect of charge modification of PICs derived from the F(ab′)$_2$ fragment of OC125 on their biodistribution in a new xenograft model of ovarian cancer. Because the effect of charge on the i.p. biodistribution of immunoconjugates is unknown, and because the use of F(ab′)$_2$ fragments shows advantages over intact IgG molecules in terms of penetration into tissue (Buchegger et al, 1990), we have developed a new conjugation strategy applicable to F(ab′)$_2$ fragments and in which the charge can be either positive or negative. The PICs bearing either polycationic or polyanionic charges were prepared, labelled with $^{125}$I and injected i.p. into a murine model derived a human

*Received 25 April 1996*
*Revised 14 October 1996*
*Accepted 21 October 1996*

*Correspondence to:* T Hasan
*The contributions of the first two authors were equal ovarian carcinoma cell line (NIH:OVCAR-5). Biodistribution of both the protein moiety and the photosensitizer moiety were measured independently via tissue content of $^{125}I$ radioactivity and $c_{e6}$ fluorescence respectively.

### MATERIALS AND METHODS

### Mice

Female Swiss nude mice (Cox Breeding Laboratories, Cambridge, MA, USA) 6–8 weeks old received proper care and maintenance according to institutional guidelines. They had continuous access to food and water, which was taken ad libitum. Throughout the experiments mice were housed in laminar flow racks under specific pathogen-free conditions and were monitored daily for general health status.

### Tumour cells

NIH:OVCAR-5 cells were purchased from Dr Tom Hamilton (Fox Chase Cancer Institute, Philadelphia, PA, USA). Cells were grown in RPMI-1640 medium supplemented with 10% heat-inactivated fetal calf serum (FCS) and 1% Hepes and were maintained in an incubator (Queue Cell Culture Incubator, model 2720; Queue Systems, Parkersburg, WV, USA) at 37°C in an atmosphere of 5% carbon dioxide. Cells were harvested for injection during the log growth phase, when growth was 80–85% confluent. For harvest and transplantation, cells were trypsinized (trypsin-EDTA; Gibco, Grand Island, NY, USA), centrifuged at 20°C for 10 min at 148 *g* (model RT6000; Sorvall Centrifuges, Dupont, Wilmington, DE, USA) and resuspended in phosphate-buffered saline without calcium or magnesium (PBS, Gibco) for counting.

### Antibodies

Monoclonal antibody OC125 $F(ab')_2$ was a kind gift from Centacor (Malvern, PA, USA) and rabbit IgG was purchased from Sigma (St Louis, MO, USA). Both proteins were radiolabelled with $^{125}I$ by the Iodogen procedure (Salacinski et al, 1981). The specific activity of the OC125F$(ab')_2$ was 8.7 MBq $mg^{-1}$, while that of the rabbit IgG was 2.1 MBq $mg^{-1}$.

### Conjugation procedure

The procedure has been described in detail elsewhere (Hamblin et al, 1996). Briefly, poly-L-lysine (average mol. wt. 25 000) was treated in dimethyl sulphoxide (DMSO) with the *N*-hydroxysuccinimide ester of $c_{e6}$ to give pl-$c_{e6}$. This was then reacted with pyridyldithiopropionic acid *N*-hydroxysuccinimide ester to form the functionalized derivative pl-$c_{e6}$-SPDP. This was then split into two parts and one part was treated with an excess of succinic anhydride to give the negatively charged functionalized pl-$c_{e6}$-succ-SPDP. [$^{125}I$]OC125F$(ab')_2$ or $^{125}I$-labelled rabbit IgG was reduced for 1 h with 5 mM mercaptoethylamine hydrochloride, dialysed and then reacted with either pl-$c_{e6}$-SPDP or pl-$c_{e6}$-succ-SPDP to form the cationic and anionic PICs respectively. When pl-$c_{e6}$ was treated with succinic anhydride, pl-$c_{e6}$-succ was obtained. The conjugates were purified by chromatography on Sephadex G200 columns and characterized by absorption and fluorescence spectrophotometry, and by polyacrylamide gel electrophoresis. The structures of the cationic PIC OC125F$(ab')_2$-pl-$c_{e6}$ and the anionic PIC OC125F$(ab')_2$-pl-$c_{e6}$-succ are shown in Figure 1.

Cationic OC125F$(ab')_2$pl-ce6 — Anionic OC125F$(ab')_2$pl-ce6-succ

**Figure 1** Chemical structures of the cationic PIC OC125F$(ab')_2$-pl-$c_{e6}$ and the anionic PIC OC125F$(ab')_2$-pl-$c_{e6}$-succ

© Cancer Research Campaign 1997

**Figure 2** Determination of immunoreactivity of MAbs and PICs by ELISA on fixed OVCAR-5 cells ■, OC125F(ab′)$_2$, □, OC125F(ab′)$_2$-pl-c$_{e6}$; ●, OC125F(ab′)$_2$-pl-c$_{e6}$-succ; ○, mouse IgG

### ELISAs

OVCAR-5 cells were grown in a 96-well plate (10 000 cells ml$^{-1}$) to which was added RPMI containing 5% FCS. Cells were fixed with 0.025% glutaraldehyde, washed three times with PBS and blocked with 1% FCS, 1% glycine and 0.02% sodium azide. The MAb, conjugate or control mouse IgG was serially diluted from 0.1 mg ml$^{-1}$ to 10 ng ml$^{-1}$ in PBS and 0.1 ml of protein solution was added and allowed to incubate for 2 h at room temperature. The MAb was removed and the fixed cells washed three times with PBS containing 0.05% Tween 20. To each well was added 0.1 ml of F(ab′)$_2$ fragment horseradish peroxidase-conjugated rabbit anti-mouse IgG (Zymed) diluted 1:400 in PBS containing 0.05% bovine serum albumin (BSA) and 0.05% Tween 20. This was allowed to incubate for 2 h, removed, washed three times with PBS/0.05% Tween 20 and 0.1 ml of a freshly prepared solution of 1,2-phenylenediamine (0.4 mg ml$^{-1}$) in 0.05 M sodium citrate, 0.15 M sodium phosphate, pH 6, containing 0.032% 30% hydrogen peroxide was added. This was allowed to incubate in the dark for 1 h, after which 0.05 ml of 4 M sulphuric acid was added to terminate the reaction. The absorbance was read at 492 nm in the automatic plate reader.

### Animal model of ovarian cancer

This has been described in detail elsewhere (Molpus et al, 1996*b*). Briefly, NIH:OVCAR-5 cells in culture were harvested as described and the pellet of cells obtained by centrifugation was suspended in PBS to a concentration of $1 \times 10^7$ cells ml$^{-1}$. Intraperitoneal injection into female nude mice was performed with $1 \times 10^7$ cells in 1 ml. Within 14 days, the animals developed small, multifocal tumour nodules throughout the peritoneal cavity. Twenty-eight days after inoculation, animals developed significant macroscopic tumour burden in the abdomen and pelvis, at which time biodistribution studies were performed.

### Biodistribution of radiolabelled OC125F(ab′)$_2$ PICs and control conjugates in tumour-bearing nude mice

Twenty-eight days after intraperitoneal injection of NIH:OVCAR-5 tumour cells, groups of 12 mice were given i.p. injections of $^{125}$I radiolabelled cationic and anionic OC125F(ab′)$_2$ PICs containing 10 µg of c$_{e6}$ conjugated to approximately 80 µg of MAb in 1 ml of sterile PBS. Also injected was unconjugated [$^{125}$I]OC125F(ab′)$_2$ (80 µg of protein), unconjugated [$^{125}$I]rabbit IgG (135 µg of protein), cationic and anionic [$^{125}$I]rabbit IgG PICs (10 µg of c$_{e6}$ bound to 135 µg of protein), non-radioactive pl-c$_{e6}$, pl-c$_{e6}$-succ and free c$_{e6}$ (10 µg of c$_{e6}$ equivalent), all in 1 ml of sterile PBS.

Six mice in each group were sacrificed using inhalation anaesthesia at 3-h and 24-h intervals. Two tumour nodules (one from the pelvis and one from the abdomen) and samples of normal tissues

**Table 1** Tumour – normal tissue ratios of $^{125}$I contained per gram of tissue and %ID per g in tumour[a] (±s.e.m.)

| $^{125}$I conjugate | Time (h) | Tumour – liver | Tumour – kidney | Tumour – intestine | Tumour – skin | Tumour – spleen | Tumour – lung | Mean ratio | %ID$^{125}$I per g tumour |
|---|---|---|---|---|---|---|---|---|---|
| OC125F(ab′)$_2$ | 3 | 5.7 ± 2.3 | 2.3 ± 0.2 | 3.5 ± 0.7 | 2.1 ± 0.6 | 1.9 ± 0.3 | 5.4 ± 2.1 | 3.5 ± 0.4 | 30.1 ± 1.3 |
| OC125F(ab′)$_2$ | 24 | 2.7 ± 0.6 | 2.8 ± 0.2 | 5.4 ± 0.5 | 3.0 ± 1.2 | 1.4 ± 0.2 | 4.0 ± 1.7 | 3.2 ± 0.4 | 38.1 ± 2.9 |
| OC125F(ab′)$_2$-pl-c$_{e6}$ | 3 | 5.4 ± 1.2 | 0.9 ± 0.1 | 6.4 ± 1.2 | 3.3 ± 0.8 | 4.7 ± 2.5 | 10.6 ± 5.2 | 5.2 ± 1.0 | 25.8 ± 6.5 |
| OC125F(ab′)$_2$-pl-c$_{e6}$ | 24 | 4.2 ± 2.5 | 1.5 ± 1.0 | 2.9 ± 1.4 | 3.6 ± 3.0 | 2.6 ± 1.5 | 3.3 ± 1.1 | 3.0 ± 0.4 | 17.1 ± 1.8 |
| OC125F(ab′)$_2$-pl-c$_{e6}$-succ | 3 | 4.9 ± 1.7 | 3.6 ± 1.8 | 3.5 ± 0.9 | 2.4 ± 0.5 | 2.4 ± 0.6 | 2.7 ± 0.5 | 3.3 ± 0.4 | 6.3 ± 1.3. |
| OC125F(ab′)$_2$-pl-c$_{e6}$-succ | 24 | 2.8 ± 0.9 | 2.4 ± 1.0 | 7.1 ± 1.7 | 1.9 ± 0.7 | 3.0 ± 1.0 | 6.0 ± 0.9 | 3.9 ± 0.7 | 3.8 ± 0.9 |
| Rabbit IgG | 3 | 2.7 ± 0.7 | 1.5 ± 0.5 | 2.6 ± 0.7 | 0.7 ± 0.2 | 1.8 ± 0.5 | 2.6 ± 1.0 | 1.8 ± 0.2 | 5.2 ± 1.6 |
| Rabbit IgG | 24 | 1.1 ± 0.5 | 1.0 ± 0.2 | 1.5 ± 0.2 | 0.7 ± 0.1 | 0.8 ± 0.3 | 0.8 ± 0.1 | 0.86 ± 0.1 | 2.9 ± 0.5 |
| Rabbit IgG-pl-c$_{e6}$ | 3 | 1.1 ± 0.4 | 1.2 ± 0.3 | 3.1 ± 0.5 | 0.7 ± 0.2 | 0.8 ± 0.3 | 1.4 ± 0.5 | 2.2 ± 0.25 | 1.9 ± 0.4 |
| Rabbit IgG-pl-c$_{e6}$ | 24 | 1.0 ± 0.2 | 1.0 ± 0.2 | 2.0 ± 0.3 | 0.4 ± 0.1 | 0.6 ± 0.1 | 0.9 ± 0.2 | 1.3 ± 0.14 | 2.0 ± 0.3 |
| Rabbit IgG-pl-c$_{e6}$-succ | 3 | 2.7 ± 0.6 | 1.5 ± 0.4 | 3.9 ± 0.7 | 0.9 ± 0.2 | 2.1 ± 0.5 | 2.8 ± 0.7 | 1.5 ± 0.1 | 6.7 ± 2.3 |
| Rabbit IgG-pl-c$_{e6}$-succ | 24 | 1.5 ± 0.3 | 1.0 ± 0.4 | 1.5 ± 0.2 | 0.7 ± 0.1 | 0.8 ± 0.2 | 1.4 ± 0.2 | 0.9 ± 0.1 | 3.9 ± 0.8 |

[a]Nude mice bearing i.p. OVCAR-5 tumours (six per time point) were injected with $1.2 \times 10^7$ c.p.m. $^{125}$I bound to OC125F(ab′)$_2$ alone or conjugated (approximately 80 µg of protein) or $5 \times 10^6$c.p.m. $^{125}$I-labelled rabbit IgG (approximately 135 µg of protein). After 3 h or 24 h, animals were sacrificed and samples of tumour and normal tissue removed, weighed and the $^{125}$I determined in a gamma counter. Values are the means of ratios calculated for each of six mice (each tumour $^{125}$I value was the mean of two separate tumour samples). Mean ratios were calculated by taking the mean of 36 tumour – normal ratios (for six organs from six mice).

© *Cancer Research Campaign 1997* 

**Figure 3** Biodistribution of $^{125}$I expressed as percentage dose per gram of tissue taken from tumour-bearing nude mice (**A**) 3 h and (**B**) 24 h after injection of $1.2 \times 10^7$c.p.m. ■, [$^{125}$I] OC125F(ab′)$_2$; □, [$^{125}$I]OC125F(ab′)$_2$-pl-$c_{e6}$; ▨, [$^{125}$I]OC125F(ab′)$_2$-pl-$c_{e6}$-succ. Values are means (± s.e.m.) from six mice (tumour mean is of two separate samples from each of six mice)

**Figure 4** Biodistribution of $^{125}$I expressed as percentage dose per gram of tissue taken from tumour-bearing nude mice (**A**) 3 h and (**B**) 24 h after injection of 8.9× $10^6$c.p.m. ■, $^{125}$I-labelled rabbit IgG; □, $^{125}$I rabbit IgG-pl-$c_{e6}$; ▨, $^{125}$I-labelled rabbit IgG-pl-$c_{e6}$-succ. Values are means (± s.e.m.) from six mice (tumour mean is of two separate samples from each of six mice)

(skin, liver, kidney, intestine, spleen and lung) were immediately removed from each mouse, weighed (wet weights were between 25 and 100 mg) and counted in a gamma counter to determine $^{125}$I activity. Following counting, all samples were lyophilized and suspended in 2 ml of 1 N sodium hydroxide containing 0.1% sodium dodecyl sulphate (SDS). Samples were allowed to dissolve in the dark for 7 days. Following this, homogenization was performed by hand as needed. The fluorescence of each sample was measured with a spectrofluorometer (Fluorolog 2; Spex Industries, Edison, NJ, USA) (excitation at 400 nm, emission scanned from 580 to 720 nm). The peak area above the background was measured and compared with that of standard solutions. The fluorescence of tissue samples from nine Ab-injected animals was used for the correction of endogenous fluorescence present in the skin and intestines. No significant endogenous fluorescence was found in tumour or other organs sampled.

### Statistics

Differences between means were analysed for statistical significance by the Student's *t*-test (*P*-values < 0.05 were considered significant). Standard errors of ratios of two means were found by calculating in quadrature.

### RESULTS

### Immunoreactivity of PICs

Although the formation of the PICs was carried out by a site-specific conjugation strategy in order to preserve immunoreactivity, the manipulation of the charge may have affected the antigen recognition characteristics of the MAb, and ELISAs with fixed cells were carried out to confirm that both the cationic and cationic PICs retained immunoreactivity. As can be seen from Figure 2, the anionic PICs had the highest affinity for fixed OVCAR-5 cells, closely followed by the unmodified OC125F(ab′)$_2$ and the anionic PIC, while the non-specific mouse IgG showed no significant binding.

© Cancer Research Campaign 1997

**Table 2** Tumour – normal tissue ratios of fluorescence and absolute tumour fluorescence extracted per gram of tissue[a] (± s.e.m.)

| $c_{e6}$ Conjugate | Time (h) | Tumour – liver | Tumour – kidney | Tumour – intestine | Tumour – skin | Mean ratio | Fluor (× $10^6$) per gram tumour |
|---|---|---|---|---|---|---|---|
| Cationic OC125F(ab′)$_2$-pl-$c_{e6}$ | 3 | 35.6 ± 15 | 13.8 ± 5.7 | 9.7 ± 5.4 | 15.8 ± 6.8 | 18.7 ± 4.5 | 23.2 ± 10.0 |
| Cationic OC125F(ab′)$_2$-pl-$c_{e6}$ | 24 | 56.7 ± 22 | 30.3 ± 19 | 0.7 ± 0.5 | 6.8 ± 3.2 | 23.1 ± 8.1 | 28.3 ± 9.6 |
| Anionic OC125F(ab′)$_2$-pl-$c_{e6}$-succ | 3 | 7.5 ± 4.8 | 5.6 ± 4.2 | 0.2 ± 0.1 | 10.1 ± 9.4 | 7.7 ± 2.6 | 3.3 ± 1.9 |
| Anionic OC125F(ab′)$_2$-pl-$c_{e6}$-succ | 24 | 36.6 ± 15 | 14.3 ± 5.3 | 4.0 ± 1.3 | 19.5 ± 17 | 18.6 ± 5.8 | 11.0 ± 5.0 |
| Cationic rabbit IgG-pl-$c_{e6}$ | 3 | 42.2 ± 30 | 36.7 ± 19 | 0.56 ± 0.3 | 8.2 ± 1.7 | 22 ± 6.4 | 7.3 ± 2.7 |
| Cationic rabbit IgG-pl-$c_{e6}$ | 24 | 62.6 ± 20 | 50 ± 21 | 1.0 ± 0.5 | 7.8 ± 3.6 | 30.4 ± 8.4 | 7.4 ± 3.0 |
| Anionic rabbit IgG-pl-$c_{e6}$-succ | 3 | 54 ± 15.7 | 20 ± 15 | 0.7 ± 0.3 | 2.3 ± 1.7 | 19.3 ± 6.6 | 3.3 ± 1.7 |
| Anionic rabbit IgG-pl-$c_{e6}$-succ | 24 | 44.4 ± 14 | 12.4 ± 8.6 | 2.2 ± 1.5 | 0.9 ± 0.3 | 18 ± 5.7 | 6.3 ± 1.6 |
| Cationic pl-$c_{e6}$ | 3 | 26 ± 12 | 9.4 ± 0.8 | 0.26 ± 05 | 4 ± 2.7 | 10 ± 2.6 | 6.5 ± 2.9 |
| Cationic pl-$c_{e6}$ | 24 | 10.7 ± 8 | 3.4 ± 2.6 | 0.06 ± .04 | 0.13 ± .06 | 3.6 ± 1.6 | 3.0 ± 1.2 |
| Anionic pl-$c_{e6}$-succ | 3 | 6.7 ± 3.2 | 54 ± 26 | 0.1 ± 0.4 | 6.2 ± 2.8 | 16.8 ± 8.3 | 3.2 ± 0.7 |
| Anionic pl-$c_{e6}$-succ | 24 | 27 ± 24 | 50 ± 27 | 0.02 ± .01 | 0.3 ± 0.2 | 19.6 ± 1.0 | 1.3 ± 0.2 |
| $c_{e6}$ | 3 | 15.7 ± 2.8 | 38 ± 23 | 0.14 ± .05 | 0.55 ± .14 | 13.7 ± 5.2 | 5.4 ± 1.0 |
| $c_{e6}$ | 24 | 51 ± 19 | >100 | 0.4 ± 0.2 | 1.9 ± 1.4 | 38.4 ± 9.2 | 9.9 ± 0.2 |

[a]Nude mice bearing i.p. OVCAR-5 tumours (six per time point) were injected with 10 µg of $c_{e6}$ equivalent bound to the specified conjugate. After 3 h or 24 h, animals were sacrificed and samples of tumour and normal tissue removed, weighed, the fluorescence extracted and the peak area of the fluorescence emission measured. Values are the means of tumour to normal ratios calculated from each of six mice (each tumour fluorescence value was the mean of two separate tumour samples). Mean ratios were calculated by taking the mean of 24 tumour – normal ratios (for four organs from six mice).

**Figure 5** Relative amounts of $c_{e6}$ fluorescence in i.p. tumour nodules taken from nude mice injected i.p. with PIC (10 µg of $c_{e6}$ equivalent) after time shown. Values are means (± s.e.m.) of 12 tumour samples (two separate tumour samples from each of six mice at each time point). ■, after 3 h; □, after 24 h

### Animal model

The predominant clinical manifestation is diffuse, macroscopic intraperitoneal disease. Tumour deposits cover all serosal surfaces especially pelvis, subgastrium and peritoneum. Widespread carcinomatosis preceded any significant ascites production. Owing to the extensive presence of macroscopic and microscopic tumour, it proved impossible to dissect areas of normal peritoneum to evaluate the uptake of the MAbs and PICs by normal peritoneum.

### Tumour: normal tissue ratios of $^{125}$I

These are shown for six normal organs and for the means of the ratios for all six organs in Table 1. Also given for comparison are the values for %ID $^{125}$I per g of tumour. Unmodified OC125F(ab′)$_2$ and rabbit IgG are compared with cationic and anionic PICs prepared from these proteins. The mean ratios obtained with OC125F(ab′)$_2$ and its cationic and anionic PICs are significantly greater (two to four times) than the corresponding mean ratios obtained with the equivalent rabbit IgG conjugate and time after injection ($P < 0.01$ in each of six cases). At 3 h, the mean ratio for cationic OC125F(ab′)$_2$-pl-$c_{e6}$ (5.2 ± 1.0) was significantly greater than that for unmodified OC125F(ab′)$_2$ at 3 h (3.5 ± 0.4, $P < 0.05$) and at 24 h (3.2 ± 0.4,$P < 0.05$) and anionic OC125F(ab′)$_2$-pl-$c_{e6}$-succ at 3 h (3.3 ± 0.4, $P < 0.05$) but not at 24 h. However, this ratio obtained with the cationic OC125F(ab′)$_2$-pl-$c_{e6}$ fell sharply at 24 h (3.0 ± 0.4, $P < 0.05$). The mean ratios for $^{125}$I delivered by rabbit IgG (both unmodified protein and PICs) were found to be significantly higher at 3 h than at 24 h ($P < 0.05$ for all three forms). In addition, at 3 h the mean ratio from the cationic rabbit IgG PIC (2.2 ± 0.25) was significantly higher than the anionic rabbit IgG PIC (1.47 ± 0.1, $P < 0.01$), but not the unmodified rabbit IgG. The main difference in organ distribution was the low tumour – kidney ratio when the $^{125}$I was delivered by the cationic OC125F(ab′)$_2$-pl-$c_{e6}$, which was significantly lower than that from unmodified OC125F(ab′)$_2$ ($P < 0.001$) and from anionic OC125F(ab′)$_2$-pl-$c_{e6}$-succ ($P < 0.05$) at 3 h.

### Percentage injected dose per gram of $^{125}$I from OC125F(ab′)$_2$ and rabbit IgG PICs

$^{125}$I uptake expressed as the percentage injected dose per gram of tissue into six normal organs and the mean of two tumour samples, delivered by unmodified OC125F(ab′)$_2$ and cationic and anionic OC125F(ab′)$_2$ PICs after 3 h is shown in Figure 3A and after 24 h in Figure 3B. It can be seen that the amount of $^{125}$I retained not only in tumour but also in normal organs is highest for unmodified OC125F(ab′)$_2$, lower for the cationic OC125F(ab′)$_2$ PIC and lowest for the anionic OC125F(ab′)$_2$ PIC at both time points. Figure 4A and B shows the percentage injected dose per gram for $^{125}$I delivered by rabbit IgG and its conjugates. The overall values are significantly lower than those found for the OC125F(ab′)$_2$ and, in addition to the values for tumour being lower, the values for skin are higher.

© Cancer Research Campaign 1997

### Endogenous fluorescence in uninjected mice

It has been reported (Weagle et al, 1988) that mice fed on laboratory chow containing chlorophyll have varying amounts of a chlorin-like metabolite in their gastrointestinal tract and sometimes in their skin. In agreement with these results, we found chlorin-like fluorescence emission spectra in tissue extracts from the intestines and skin of non-injected mice. These values were variable and generally lower than that delivered to these organs by i.p. injected $c_{e6}$ derivatives, and the mean values of endogenous fluorescence peak areas per gram of tissue from nine control mice were subtracted from the peak areas per gram of skin and intestine tissue in the mice injected with $c_{e6}$ conjugates.

### Tumour–normal tissue ratios of $c_{e6}$

The tumour to normal tissue ratios of $c_{e6}$ content measured for four normal organs (liver, kidney, intestines and skin) and the means of these four ratios, removed at 3-h and 24-h intervals after i.p. injection, are shown in Table 2. Also given for comparison are the absolute fluorescence content for the tumour in peak area per gram. These are given for $c_{e6}$ delivered by the cationic and anionic charged forms of OC125F(ab′)$_2$ PIC, rabbit IgG PIC, polylysine conjugates and free $c_{e6}$. Values are not given for the tumour to normal ratios of spleen and lung, as the amount of fluorescence extracted from these tissues was so low that the ratios would have been unreasonably high (from 100–10 000). The mean of the tumour: normal ratios at 3 h obtained from the cationic OC125F(ab′)$_2$ PIC (18.7 ± 4.5) was significantly higher than that from the anionic OC125F(ab′)$_2$PIC (7.7 ± 2.6, $P < 0.05$), significantly higher than cationic pl-$c_{e6}$ (10.0 ± 2.0, $P < 0.05$), but not different from cationic rabbit IgG-pl-$c_{e6}$. At 24 h, the ratio from the cationic OC125F(ab′)$_2$ PIC (23.1 ± 8.1) was not significantly different from the anionic OC125(ab′)$_2$ PIC (18.6 ± 5.8) or from the cationic rabbit IgG-pl-$c_{e6}$ (30.4 ± 8.4), but was significantly higher than cationic pl-$c_{e6}$ (3.6 ± 1.6, $P < 0.01$). The mean ratio from the anionic OC125F(ab′)$_2$PIC at 24 h (18.6 ± 5.8) was significantly greater than that found from the same PIC at 3 h (7.7 ± 2.6, $P < 0.05$), but neither at 3 h nor 24 h did the anionic OC125F(ab′)$_2$ PIC give significantly higher tumour to normal ratios than the anionic rabbit IgG PIC or the anionic pl-$c_{e6}$-succ.

**Figure 6** Mean values (± s.e.m.) of percentage injected dose of $^{125}$I per gram of tissue calculated as means of the six values for normal organs and mean value for two tumour samples from Figures 2 and 3. ■, after 3 h; □, after 24 h

It should be noted that one of the most sensitive normal structures to i.p. PDT is the intestines (Veenhuizen et al, 1994). In this case, the tumour to intestine ratios for the cationic OC125F(ab′)$_2$ PIC at 3 h (9.7 ± 3.4) and the anionic OC125F(ab′)$_2$ PIC at 24 h (4.0 ± 1.3) were significantly greater than the tumour to intestines ratios of all species at all time points ($P < 0.05$) except the anionic rabbit IgG PIC at 24 h (2.2 ± 1.5).

### Relative amount of $c_{e6}$ delivered to tumour by OC125F(ab′)$_2$ and rabbit IgG PICs

Although the tumour to normal ratios obtained with $c_{e6}$ delivered by OC125F(ab′)$_2$ PICs were not significantly higher than those found with rabbit IgG PICs for either charge or time point, there may still be an advantage in using OC125F(ab′)$_2$ PICs, if they deliver a higher amount of the PS to the tumour. That this is indeed so for the cationic conjugates is shown in Figure 5. The uptake from the OC125F(ab′)$_2$ cationic PIC was 3.2 times greater at 3 h and 3.8 times greater at 24 h than that from the rabbit IgG cationic PIC ($P < 0.05$ in both cases). The uptake of $c_{e6}$ delivered by the cationic OC125F(ab′)$_2$ PIC was also 7.6 times and 2.5 times higher than that from the anionic OC125F(ab′)$_2$ PIC at 3 h and 24 h respectively ($P < 0.05$ in both cases). Aside from the cationic OC125F(ab′)$_2$ PIC, other species did not give significant differences except for the anionic OC125F(ab′)$_2$ PIC, which at 24 h delivered 33 times more than anionic pl-$c_{e6}$-succ ($P < 0.01$).

### DISCUSSION

Conjugation procedures may, in addition to sometimes reducing the affinity and or the specificity of the MAb for the antigen, produce structural changes so profound that the fundamental biochemical trafficking of the MAb is changed. This change may be either for the better or for the worse, as far as targeting the cytotoxic moiety to the tumour is concerned.

The present study has investigated the biodistribution of OC125F(ab′)$_2$ PICs of opposite charge after i.p. administration in an i.p. xenograft model of ovarian cancer in nude mice. The biodistribution of $^{125}$I-labelled protein was measured independently from the PS $c_{e6}$. There are three biodistribution parameters that may affect the efficacy of i.p. PDT. These are (1) the maximum concentration of the PS in the tumour; (2) the maximum tumour – normal tissue ratio of PS with respect to the most sensitive normal structure (thought to be the intestines; (Veenhuizen et al, 1994); and (3) the maximum penetration depth of the PS into the tumour nodules. This study has not addressed point (3) at all, and has gained preliminary data on parameters (1) and (2) with two opposite charged PICs and two time points after administration.

The cationic OC125F(ab′)$_2$ PIC has a marked advantage in delivering a higher amount of $c_{e6}$ to the tumour than the anionic PIC, especially at 3 h. In addition, the cationic OC125F(ab′)$_2$ PIC delivers more $c_{e6}$ (2.5–80 times) than any other species at both time points. The cationic rabbit PIC also produces tumour–normal tissue ratios that are remarkably similar to those produced by the cationic OC125F(ab′)$_2$ PIC at both time points, but the absolute amount of $c_{e6}$ delivered to the tumour was 3.2 and 3.8 times higher for the cationic OC125F(ab′)$_2$ PIC at 3 h and 24 h respectively. The cationic OC125F(ab′)$_2$ PIC is also the only delivery vehicle, which gives sizeable tumour to intestine ratios of $c_{e6}$. It is thought (Veenhuizen et al, 1994) that the intestines are the most sensitive normal intraperitoneal organ to phototoxicity caused during i.p. PDT.

© Cancer Research Campaign 1997

It is known that imparting a pronounced positive charge to macromolecules, including monoclonal antibodies, encourages their binding to the cell and their rapid internalization (Ryser et al, 1982). Molecules that are internalized into cells by virtue of their positive charge undergo endosomal processing (Hansen et al, 1993) and accumulate in lysosomes, where proteolysis may take place. Pardridge et al (1994*a,b*) have compared unmodified and cationic monoclonal antibodies and found much increased endocytosis of the cationic species. In vivo, it has been shown that cationized IgG molecules have a much higher organ take-up than the unmodified form (Triguero et al, 1991).

By comparing the results gained from the $c_{e6}$ and $^{125}I$ measurements, it is possible to gain insights into the extent to which the MAb is responsible for the tumour targeting, and what is happening to the constituent parts of the PIC. The data show a marked separation of the $c_{e6}$ from the $^{125}I$. The $c_{e6}$ is much more likely to stay in the tissues directly exposed to peritoneal fluid, while the $^{125}I$ is more likely to travel to distant organs via the bloodstream or the lymphatics. The extremely low levels of $c_{e6}$ found in the lungs and spleen indicate that very little $c_{e6}$ reaches the bloodstream compared with $^{125}I$. There are three possible ways in which this separation could happen. Firstly, PICs could be endocytosed into lysosomes and proteolysed to small peptides and amino acids, which could then leave the cells and enter the circulation. This has been demonstrated to occur in vitro (Press et al, 1988) and is thought to be the most likely mechanism in vivo (Press et al, 1990). Secondly, the $^{125}I$ could be removed from the protein by a deiodinating activity (Smith et al, 1993, 1994), and the free iodide ion could diffuse rapidly throughout the circulation. Thirdly, the pl-$c_{e6}$ and pl-$c_{e6}$-succ are attached to the OC125F(ab′)$_2$ via a non-hindered disulphide bond that has been shown to be susceptible to in vivo reduction (Carroll et al, 1994). This would separate the $c_{e6}$ from the $^{125}I$ but would leave the latter still in the OC125F(ab′)$_2$, which might then be expected to give higher tumour to normal ratios of $^{125}I$ than $c_{e6}$, which is the opposite of what is found. If the cationic conjugates are taken up by i.p. tumour cells via endocytosis and degraded in lysosomes where the $^{125}I$ was released and the $c_{e6}$ retained, this would explain the higher tumour to normal ratios of $c_{e6}$ compared with $^{125}I$, and would also be consistent with in vitro data using these OC125F(ab)$_2$ PICs (Hamblin et al, 1996) and previous work using conjugates between$^{125}I$ labelled low-density lipoprotein and haematoporphyrin (Hamblin and Newman, 1994). The high ratios but low absolute content of $c_{e6}$ found for the cationic and anionic rabbit IgG PICs are also consistent with this hypothesis. However, it must be stated that the rabbit IgG PICs were formed from an intact IgG not a F(ab′)$_2$ fragment, and this may lead to different pharmacokinetic parameters. The in vivo metabolism of the PICs could be confirmed by carrying out experiments that looked at the degree to which $c_{e6}$ and $^{125}I$ in various tissue extracts could be precipitated by trichloroacetic acid.

The mean values of percentage injected dose $^{125}I$ per gram of tissue for six normal organs and two tumour samples are shown in Figure 6. It is apparent that there are significant differences between PICs in the extent to which $^{125}I$ is taken up into organs. Unmodified OC125F(ab′)$_2$ has an uptake that is twice as high ($P <$ 0.01) as cationic OC125F(ab′)$_2$ PIC at both time points, while this uptake in turn is three times ($P < 0.005$) higher than that from the anionic OC125F(ab′)$_2$ PIC. These differences are not apparent with the uptake from the rabbit IgG PICs, which seem to deliver the same percentages of injected dose to tissues regardless of the charge on the PIC. There is therefore some difference between the unmodified OC125F(ab′)$_2$ PIC and rabbit IgG that causes the former to be taken up into tissue much more and, in addition, makes variation in the charge of OC125F(ab′)$_2$ affect the degree to which it is taken up into tissue. This difference may either be the fact of antibody antigen recognition or the fact that the MAb is a F(ab′)$_2$ fragment, while the rabbit is an entire IgG molecule. If the cationic OC125F(ab′)$_2$ PIC is endocytosed and subject to lysosomal hydrolysis, this explains the higher uptake of $c_{e6}$ (compared with the anionic) by the tumour, the higher tissue uptake of $^{125}I$ and also the higher uptake of $^{125}I$ by the kidneys (without concomitant $c_{e6}$), as proteolytic fragments and amino acids are small enough to be excreted by glomerular filtration (Smith et al, 1993). The high tissue uptake of the unmodified OC125F(ab′)$_2$, however, may not be by endocytosis and hydrolysis, but by transcytosis across the mesothelial barrier of the peritoneum (Flessner and Dedrick, 1994) or via diaphragmatic lymphatics (Abernethy et al, 1991) into the circulation and hence to tumour and other organs. The low uptake of anionic OC125F(ab′)$_2$ is presumably explained by the negative charge inhibiting the uptake found with unmodified OC125F(ab′)$_2$, and not stimulating endocytosis as seen with the cationic PIC.

The fact that the tumour–normal ratios of $^{125}I$ are higher for the OC125F(ab′)$_2$ of both charges than they are for the equivalent rabbit IgG PICs while the ratios of $c_{e6}$ are similar, can be explained if one assumes that a PIC that binds to a cell owing to MAb antigen attraction is less likely to be internalized than the same PIC that binds via charge interaction. Then a greater proportion of the cationic rabbit IgG PIC that can only bind via charge will be internalized, degraded and the $^{125}I$ lost from the tumour tissue than is found for the cationic OC125F(ab′)$_2$-pl-$c_{e6}$

The mean tumour–normal (blood, liver, kidney, intestine and spleen) ratios found in the study by Thedrez et al (1989) using $^{111}In$-labelled intact OC125 injected i.p. into nude mice bearing OVCAR-3 tumours were higher than those found at similar time points with our [$^{125}I$]OC125F(ab′)$_2$ (at 3 h $6.0 \pm 1.4$ vs $3.5 \pm 0.4$ and at 24 h $6.9 \pm 2.7$ vs $3.2 \pm 0.4$ respectively). The reasons for this difference may be the decreased metabolism of $^{111}In$-labelled MAbs, the use of intact IgG or the use of OVCAR-3 cells, which express more CA125 than OVCAR-5 cells. However, the tumour to normal ratio of $^{125}I$ obtained by the cationic PIC at 3 h ($5.2 \pm 1.0$) is similar to the value found with the $^{111}In$-labelled MAb ($6.0 \pm 1.4$).

In summary, this initial biodistribution study demonstrates a distinct advantage of administering PICs with a positive charge to i.p. xenograft model of ovarian cancer. For example, the mean tumour – normal (blood, liver, kidney, skin and intestine) ratios reported by Goff et al (1994) for the PS (CMA) delivered by intact OC125-PGA-CMA were at $6.8 \pm 1.4$ at 3h and $5.9 \pm 0.7$ at 24 h, which are much lower than those found in the present case ($18.7 \pm 4.5$ and $23.1 \pm 8.1$ respectively). For comparison, ratios found with non-conjugated PS using HpD injected i.p. into mice bearing i.p. teratoma (Tochner et al, 1986) were $9.3 \pm 1.4$ at 2 h and $2.4 \pm 1.0$ at 24 h (tumour: liver, kidney, intestine and muscle), and, using the present model and i.p. injected liposomal benzoporphyrin derivative (Molpus et al, 1996*a*), $1.4 \pm 0.4$ at 2 h and $2.1 \pm 1.0$ at 24 h (tumour – liver, kidney, skin, intestine, peritoneum and spleen). The tumoricidal efficiency of the $c_{e6}$ will be determined by, among other factors, whether the photochemistry of $c_{e6}$ has been affected by the conjugation, and on the macroscopic and microscopic site(s) of localization of the PICs. These questions are under investigation in our laboratory.

© Cancer Research Campaign 1997

### ACKNOWLEDGEMENTS

This work was supported by a grant from the National Institute of Health (R01 AR40352) and in part (services) by the DoD FEL program. We thank Centocor for the gift of OC125F(ab')$_2$ and Dr K Molpus for his contribution in developing the xenograft model.

### REFERENCES

Abernethy NJ, Chin W, Hay JB, Rodela H, Oreopoulos D and Johnston MG (1991) Lymphatic drainage of the peritoneal cavity in sheep. *Am J Physiol* **260**: F353–F358

Beck E, Hofmann M, Bernhardt G, Jager W, Wildt L and Lang N (1994) *In vitro* activity of immunoconjugates between cisplatin and an anti-CA125 monoclonal antibody on ovarian cancer cell lines. *Cell Biophys* **25**: 163–173

Buchegger F, Pelegrin A, Delaloye B, Bischof-Delaloye A and Mach JP (1990) Iodine-131-labeled MAb F(ab')$_2$ fragments are more efficient and less toxic than intact anti-CEA antibodies in radioimmunotherapy of large human colon carcinoma grafted in nude mice. *J Nucl Med* **31**: 1035–1044

Carroll SF, Bernhard SL, Goff DA, Bauer RJ, Leach W and Kung AH (1994) Enhanced stability in vitro and in vivo of immunoconjugates prepared with 5-methyl-2-iminothiolane. *Bioconjug Chem* **5**: 248–256

Delaney TF, Sindelar WF, Tochner Z, Smith PD, Friauf WS, Thomas G, Dachowski L, Cole JW, Steinberg SM and Glatstein E (1993) Phase I study of debulking surgery and photodynamic therapy for disseminated intraperitoneal tumours. *Int J Radiat Oncol Biol Phys* **25**: 445–457

Fisher AM, Murphree AL and Gomer CJ (1995) Clinical and preclinical photodynamic therapy. *Lasers Surg Med* **17**: 2–31

Flessner MF and Dedrick RL (1994) Monoclonal antibody delivery to intraperitoneal tumors in rats: effects of route of administration and intraperitoneal solution osmolality. *Cancer Res* **54**: 4376–4384

Goff BA, Bamberg M and Hasan T (1991) Photoimmunotherapy of human ovarian carcinoma cells *ex vivo*. *Cancer Res* **51**: 4762–4767

Goff BA, Bamberg M and Hasan T (1992) Experimental photodynamic treatment of ovarian carcinoma cells with immunoconjugates. *Antibody Immunoconj Radiopharm* **5**: 191–199

Goff BA, Hermanto U, Rumbaugh J, Blake J, Bamberg M and Hasan T (1994) Photoimmunotherapy and biodistribution with an OC125-chlorin immunoconjugate in an *in vivo* murine ovarian cancer model. *Br J Cancer* **70**: 474–480

Haisma HJ, Moseley KR, Battaile AI, Griffiths TC, Zurawski VR and Knapp RC (1988) Biodistribution, pharmacokinetics and imaging of 131I-labelled OC125 in ovarian cancer. *Int J Cancer* **2** (suppl.): 109–113

Hamblin MR, Miller JL and Hasan T (1996) The effect of charge on the interaction of site-specific photosensitizer immunoconjugates with human ovarian cancer cells. *Cancer Res* **56**: 5205–5210

Hamblin MR and Newman EL (1994) Photosensitizer targeting in photodynamic therapy. II. Conjugates of haematoporphyrin with serum lipoproteins. *J Photochem Photobiol B* **26**: 147–157

Hansen SH, Sandvig K and Van Deurs B (1993) Molecules internalized by clathrin-independent endocytosis are delivered to endosomes containing transferrin receptors. *J Cell Biol* **123**: 89–97

Hasan T (1992) Photosensitizer delivery mediated by macromolecular carrier systems. In *Photodynamic Therapy: Basic Principles and Clinical Applications*, Henderson B and Dougherty T (eds) pp. 187–200. Marcel Dekker: New York

Hasan T and Parrish JA (1996) Photodynamic therapy of cancer. In *Cancer Medicine*, 4th edn, Holland JF, Frei E, Bast RC, Kufe DW, Morton DL and Weichselbaum RR(eds.), pp. 739–751. Williams & Wilkins: Baltimore (in press)

Henderson BW and Dougherty TJ (1992) How does photodynamic therapy work? *Photochem Photobiol* **55**: 145–157

Hosono MN, Endo K, Sakahara H, Watanabe Y, Saga T, Nakai T, Hosono M, Nakajima T, Onoyama Y and Konishi J (1992) Different antigenic nature in apparently healthy women with high serum CA 125 levels compared with typical patients with ovarian cancer. *Cancer* **70**: 2851–2856

Karlan BY, Amin W, Casper SE and Littlefield BA (1988) Hormonal regulation of CA125 tumor marker expression in human ovarian carcinoma cells: inhibition by glucocorticoids. *Cancer Res*, **48**: 3502–3506

Molpus KL, Kato D, Hamblin MR, Lilge L, Bamberg M and Hasan T (1996*a*) Intraperitoneal photodynamic therapy of human epithelial ovarian carcinomatosis in a xenograft murine model. *Cancer Res*, **56**: 1075–1082

Molpus KL, Koelliker D, Atkins L, Kato D, Buczek-Thomas J, Fuller AFJ and Hasan T (1996*b*) Characterization of a xenograft model of human ovarian carcinoma which produces intraperitoneal carcinomatosis and metastases in mice. *Int J Cancer* **67**: 588–595

Muto MG, Finkler NJ, Kassis AI, Howes AE, Anderson LL, Lau CC, Zurawski VR, Jr, Weadock K, Tumeh SS, Lavin P and Knapp RC (1992) Intraperitoneal radioimmunotherapy of refractory ovarian carcinoma utilizing iodine-131-labeled monoclonal antibody OC125. *Gynecol Oncol*, **45**: 265–272

Ozols RF (1995) Carboplatin and paclitaxel in ovarian cancer. *Semin Oncol*, **22**: 78–83

Pardridge WM, Bickel U, Buciak J, Yang J and Diagne A (1994*a*) Enhanced endocytosis and anti-human immunodeficiency virus type 1 activity of anti-rev antibodies after cationization. *J Infect Dis*, **169**: 55–61

Pardridge WM, Bickel U, Buciak J, Yang J, Diagne A and Aepinus C (1994*b*) Cationization of a monoclonal antibody to the human immunodeficiency virus REV protein enhances cellular uptake but does not impair antigen binding of the antibody. *Immunol Lett* **42**: 191–195

Parker SE, Tong T, Bolden S and Wingo PA (1996) Cancer statistics 1996. *CA Cancer J Clin*, **46**: 5–28

Press OW, Hansen JA, Farr A and Martin PJ (1988) Endocytosis and degradation of murine anti-human CD3 monoclonal antibodies by normal and malignant T-lymphocytes. *Cancer Res*, **48**: 2249–2257

Press OW, Desantes K, Anderson SK and Geissler F (1990) Inhibition of catabolism of radiolabeled antibodies by tumor cells using lysosomotropic amines and carboxylic ionophores. *Cancer Res*, **50**: 1243–50

Ryser HJ, Drummond I and Shen WC (1982) The cellular uptake of horseradish peroxidase and its poly(lysine) conjugate by cultured fibroblasts is qualitatively similar despite a 900-fold difference in rate. *J Cell Physiol*, **113**: 167–178

Salacinski PR, Mclean C, Sykes JE, Clement-Jones VV and Lowry PJ (1981) Iodination of proteins, glycoproteins, and peptides using a solid-phase oxidizing agent, 1,3,4,6-tetrachloro-3 alpha, 6 alpha-diphenyl glycoluril (Iodogen). *Anal Biochem*, **117**: 136–146

Smith A, Alberto R, Blaeuenstein P, Novak-Hofer I, Maecke HR and Schubiger PA (1993) Preclinical evaluation of 67Cu-labeled intact and fragmented anti-colon carcinoma monoclonal antibody MAb35. *Cancer Res*, **53**: 5727–5733

Smith A, Alberto R and Shubiger PA (1994) Influence of radiolabel on the *in vivo* processing of intact and fragmented anti-tumour monoclonal antibody. *J Nucl Biol Med* **38**: 54–58

Thedrez P, Sacca Vini JC, Nolibe D, Simoen JP, Guerreau D, Gestin JF, Kremer M and Chatal JF (1989) Biodistribution of indium-111-labeled OC 125 monoclonal antibody after intraperitoneal injection in nude mice intraperitoneally grafted with ovarian carcinoma. *Cancer Res*, **49**: 3081–3086

Tochner Z, Mitchell JB, Smith P, Harrington F, Glatstein E, Russo D and Russo A (1986) Photodynamic therapy of ascites tumours within the peritoneal cavity. *Br J Cancer* **53**: 733–736

Triguero D, Buciak JL and Pardridge WM (1991) Cationization of immunoglobulin G results in enhanced organ uptake of the protein after intravenous administration in rats and primate. *J Pharmacol Exp Ther* **258**: 186–192

Veenhuizen RB, Ruevekamp-Helmers MC, Helmerhorst TJ, Kenemans P, Mooi WJ, Marijnissen JP and Stewart FA (1994) Intraperitoneal photodynamic therapy in the rat: comparison of toxicity profiles for photofrin and MTHPC. *Int J Cancer* **59**: 830–836

Weagle G, Paterson PE, Kennedy J and Pottier R (1988) The nature of the chromophore responsible for naturally occurring fluorescence in mouse skin. *J Photochem Photobiol B* **2**: 313–320

Yarmush ML, Thorpe WP, Strong L, Rakestraw SL, Toner M and Tompkins RG (1993) Antibody targeted photolysis. *Crit Rev Ther Drug Carrier Syst* **10**: 197–252

© Cancer Research Campaign 1997